# Outbreak of KPC-2-producing *Klebsiella pneumoniae* endowed with ceftazidime-avibactam resistance mediated through a VEB-1-mutant (VEB-25), Greece, September to October 2019

**DOI:** 10.2807/1560-7917.ES.2020.25.3.2000028

**Published:** 2020-01-23

**Authors:** Irene Galani, Ilias Karaiskos, Maria Souli, Vassiliki Papoutsaki, Lamprini Galani, Aikaterini Gkoufa, Anastasia Antoniadou, Helen Giamarellou

**Affiliations:** 1 Infectious Diseases Laboratory, 4th Department of Internal Medicine, National and Kapodistrian University of Athens, Athens, Greece; 2 1^st^ Internal Medicine & Infectious Diseases Department, Hygeia General Hospital, Athens, Greece; 3 Infectious Diseases Laboratory, Hygeia General Hospital, Athens, Greece

**Keywords:** ceftazidime-avibactam, *K.pneumoniae*, KPC-2, VEB-14, VEB-25, K234R

## Abstract

From September to October 2019, seven patients colonised or infected with a ceftazidime-avibactam (CZA)-resistant *Klebsiella pneumoniae* carbapenemase (KPC)-2-producing *K. pneumoniae* were detected in two intensive care units of a Greek general hospital. The outbreak strain was sequence type (ST)147 and co-produced KPC-2 and the novel plasmid-borne Vietnamese extended-spectrum β-lactamase (VEB)-25 harbouring a K234R substitution associated with CZA resistance. Epidemiological investigations revealed that the resistance was probably acquired by horizontal transmission independently from previous CZA exposure.

The spread of carbapenemase-producing *Klebsiella pneumoniae* (CPKP) has become a significant problem worldwide. Ceftazidime-avibactam (CZA) is a novel β-lactam/β-lactamase inhibitor combination effective against strains producing serine carbapenemases, including *K. pneumoniae* carbapenemase (KPC)- and oxacillinase (OXA)-48-type enzymes [1]. Resistance to CZA has already been described and was mainly linked to specific mutations in *bla*_KPC_ [2], alterations in Ompk35 and Ompk36 porins and/or increased expression of *bla*_KPC_ [3], as well as amino acid substitutions in cefotaxime-M β-lactamase (CTX-M)-14 [4]. Moreover during editing of this report, two *K. pneumoniae* isolates resistant to CZA were reported from Greece due to Vietnamese extended-spectrum β-lactamase (VEB)-25 [5]. The European Centre for Disease Prevention and Control (ECDC) has identified CZA resistance as an important cross-border threat that merits careful monitoring [6]. In this article, we report an outbreak caused by a CZA-resistant *K. pneumoniae* strain in a hospital in Athens, Greece in 2019. The outbreak molecular investigation revealed that resistance was due to plasmid-borne VEB-25, which differs from VEB-1 by one mutation. The affected patients had not previously been treated with CZA. Another isolate detected in the same hospital in 2018 had a different *bla*_VEB-1_ variant leading to CZA-resistance but this variant did not belong to the outbreak clone.

## Outbreak detection and investigation

The index hospital comprises two mixed intensive care units (ICU) and one high dependency unit (HDU) that are located in different areas of the hospital. ICU-1 includes a 14-bed open space unit and four single-patient rooms. ICU-2 consists of seven single-patient rooms, whereas HDU has 12 beds, and one isolation room. Rectum surveillance cultures are performed routinely twice weekly in all ICU patients and screened for carbapenemase producers. In November 2018, 11 months after the introduction of CZA in clinical practice (December 2017), one patient hospitalised in the HDU was found to be colonised by a CZA-resistant KPC-producing *K. pneumoniae* strain (KP121) after CZA treatment (Table 1 and Table 2). The patient was isolated in a single room under strict contact precautions until discharge and no more cases were detected. Since then, all surveillance strains that were KPC-producers have been routinely evaluated for CZA resistance. This practice enabled the early detection of the outbreak. From September 2019 to October 2019, four patients in ICU-1 and three patients in ICU-2 were found to be colonised by a CZA-resistant KPC-producing *K. pneumoniae* strain and three of them developed an infection (Table 2). The outbreak prompted an epidemiological and molecular investigation. Prevention efforts including intensification of contact precautions (provision of personal protective equipment outside the patient room and use of gloves and gown upon entering the room, minimising risk of environmental contamination by dressing patients in a gown during transport and applying all standard precautions at the receiving unit, dedicating noncritical items for single patient use and having dedicated infection control nurses overseeing strict implementation of measures), isolation of colonised patients in single rooms and strict hand hygiene practices successfully contained the outbreak as no new case was identified after October 2019.

**Table 1:** Characteristics of the ceftazidime-avibactam-resistant *Klebsiella pneumoniae* strains detected in a general hospital^a^ as well as their transconjugants and one previously characterised transconjugant producing VEB-1, Greece, 2018 and 2019 (n = 6 strains)

Isolate	*K. pneumoniae* ST39 strain (KP121)	*E. coli* ^b^ RC85-pl121	*K. pneumoniae* ST147 outbreak strain (KP67585)	*E. coli* ^c^ RC85-pl67585	*E. coli* ^d^ RC85-pl52	*E. coli* **RC85
**Type**						
KPC-type	KPC-2	None	KPC-2	None	None	None
VEB-type	VEB-14	VEB-14	VEB-25	VEB-25	VEB-1	None
**Antibiotics tested**	**Minimum inhibitory concentration in mg/L**					
Ampicillin-sulbactam	> 16	> 16	> 16	> 16	> 16	≤ 2
Piperacillin-tazobactam	> 64	32	> 64	> 64	> 64	≤ 4
Cefoxitin	> 32	≤ 4	> 32	≤ 4	≤ 4	≤ 4
Ceftazidime	2,048	4,096	1,024	512	512	0.25
Ceftazidime-avibactam	64	256	64	16	0.25	0.25
Ceftriaxone	> 32	32	> 32	8	32	≤ 1
Cefepime	> 32	> 32	> 32	2	2	≤ 1
Aztreonam	> 32	> 32	> 32	> 32	> 32	≤ 1
Imipenem	64	0.12	32	0.12	0.12	0.12
Imipenem-relebactam	0.5	0.12	0.5	0.12	0.12	0.12
Meropenem	> 64	0.06	64	0.06	0.06	0.06
Meropenem-vaborbactam	0.5	0.06	0.25	0.06	0.06	0.06
Amikacin	> 32	> 32	> 32	> 32	> 32	≤ 2
Gentamicin	> 8	> 8	> 8	> 8	> 8	≤ 1
Ciprofloxacin	> 2	≤ 0.25	> 2	≤ 0.25	≤ 0.25	≤ 0.25
Levofloxacin	> 4	≤ 0.12	> 4	≤ 0.12	≤ 0.12	≤ 0.12
Tigecycline	> 4	1	2- > 4	2	≤ 0.5	≤ 0.5
Fosfomycin	64	≤ 16	128	≤ 16	≤ 16	≤ 16
Colistin	2	0.5	64	0.5	0.5	0.5
Trimethoprim-sulfamethoxazole^e^	> 8	> 8	> 8	> 8	> 8	≤ 1
Chloramphenicol	> 128	128	> 128	32	32	8
**Other β-lactamase genes**						
β-lactamase genes	*bla*_SHV-11,_ *bla*_OXA-10,_ *bla*_TEM-1B_	*bla*_OXA-10,_ *bla*_TEM-1B_	*bla*_SHV-11,_ *bla*_OXA-10,_ *bla*_TEM-1B_	*bla*_OXA-10,_ *bla*_TEM-1B_	*bla*_OXA-10,_ *bla*_TEM-1B_	None
**Major porin mutation**						
OmpK35	WT	ND	PSC_aa173	ND	ND	ND
OmpK36	v3 variant	ND	v3 variant	ND	ND	ND
OmpK37	PSC_aa251	ND	WT	ND	ND	ND

*E. coli*: *Escherichia coli*; *K. pneumoniae*: *Klebsiella pneumoniae;* KPC: *K. pneumoniae* carbapenemase; MIC: minimum inhibitory concentration; ND: not determined; OmpK35: OmpK35-WT (GenBank accession number: GU460162); OmpK36: OmpK36_v3 (GenBank accession number: JQ781655); OmpK37: OmpK37-WT (GenBank accession number: WP_002902433); PSC: premature stop codon; PSC_aa173: premature stop codon at amino acid 173; PSC_aa251: premature stop codon at amino acid 251; ST: sequence type; VEB: Vietnamese extended-spectrum β-lactamase; WT: wild type.

^a^ The isolates detected in the general hospital, which are presented in the table, include one isolate (KP121) producing VEB-14 (ST39) that was discovered in 2018, as well as one isolate (KP67585) producing VEB-25 (ST147) that represents a strain responsible for the 2019 hospital outbreak, which is described in this report.

^b^ Transconjugant *E. coli* RC85 isolate harbouring *bla*_VEB-14 _carrying plasmid.

^c^ Transconjugant *E. coli* RC85 isolate harbouring *bla*_VEB-25 _carrying plasmid.

^d^ Transconjugant *E. coli* RC85 isolate harbouring *bla*_VEB-1_ carrying plasmid. The donor isolate was an OXA-48-producing *K.pneumoniae* isolate carrying an IncA/C2 plasmid.

^e^ Trimethoprim-sulfamethoxazole in the ratio 1:19. MICs are expressed as the trimethoprim concentration.

**Table 2:** Demographic and clinical characteristics, outcome and follow-up of the eight patients who were colonised or infected with a ceftazidime-avibactam-resistant *Klebsiella pneumoniae* strain, Greece, 2018 (n = 1 patient) and 2019 (n = 7 outbreak cases)

Patient number (isolate name)	Approximate age in years^a^	Deptartment	Reason for ICU admission	Duration of stay in ICU/days before colonisation	Comorbidities; interventions	Colonisation^b^ date	Previous antibiotics before colonisation	Previous carbapenem use (duration in days)	Type of infection from colonised strain	Antimicrobial treatment (number of days of treatment)	Treatment outcome	Overall outcome
**1** **(KP121)**	50	HDU	SAH	153/35	Tracheostomy; MV	1 Nov 2018	CZA, MEM, TGC, CST	Yes (10) CZA (15)	No	NA	NA	Discharged to rehabilitation centre
**2** **(KP67585)**	85	ICU-2	Brain injury- subdural haematoma	107/51	CAD; tracheostomy; MV	2 Sep 2019	TZP, MEM, CIP	Yes (3)	CRBSI	CZA + MEM + FOS (15)	Success	Death^c^
**3** **(KP374)**	85	ICU-1	Thoracotomy	51/42	Metastatic malignancy; tracheostomy; MV	5 Sep 2019	MEM, CST,TGC	Yes (15)	VAP	CZA + ATM + FOS (15)	Success	Death^c^
**4** **(KP368)**	65	ICU-2	SAH	89/56	Tracheostomy; gastrostomy; MV	5 Sep 2019	TZP, AMK, VAN	No (NA)	No	NA	NA	Discharged to rehabilitation centre
**5** **(KP501)**	75	ICU-2	SAH	42/21	AH; tracheostomy; MV	16 Sep 2019	CRO, TZP, VAN	No (NA)	No	NA	NA	Discharged to rehabilitation centre
**6** **(KP687)**	70	ICU-1	Acute coronary syndrome	45/21	Renal failure; CVVHDF	26 Sep 2019	CRO, CPT, TZP, VAN, MEM	Yes (4)	CRBSI	CZA + MEM (2)	Failure	Death^d^
**7** **(KP785)**	60	ICU-1	Respiratory failure	30/11	Metastatic malignancy COPD; AH; tracheostomy; MV	3 Oct 2019	TZP, MEM, VAN	Yes (10)	No	NA	NA	Death^c^
**8** **(KP842)**	55	ICU-1	Acute coronary syndrome	12/12	AH	7 Oct 2019	CRO	No (NA)	No	NA	NA	Discharged

AH: arterial hypertension; AMK: amikacin; ATM: aztreonam; CAD: coronary arterial disease; CVVHDF: continuous veno-venous haemodiafiltration; CZA: ceftazidime-avibactam; CIP: ciprofloxacin; COPD: chronic obstructive pulmonary disease; CPT: ceftaroline; CRBSI: catheter related blood stream infection; CRO: ceftriaxone; CST: colistin; FOS: fosfomycin; HDU: high dependency unit; ICU: intensive care unit; MEM: meropenem; MV: mechanical ventilation; NA: not applicable; SAH: subarachnoid haemorrhage; TGC: tigecycline; TZP: piperacillin-tazobactam; VAN: vancomycin; VAP: ventilator-associated pneumonia.

^a^ Ages are rounded to the upper limit of 5-year categories.

^b^ Colonisation determined from analysis of a rectal sample.

^c^ Death due to another cause than *Klebsiella pneumoniae* carbapenemase *K. pneumoniae*.

^d^ Death due to bacteraemia and the patient was positive for *Klebsiella pneumoniae* carbapenemase *K. pneumoniae* infection.

## Microbiological and molecular analyses

Susceptibility testing performed by VITEK 2, broth microdilution and minimum inhibitory concentration (MIC) test strips revealed resistance, according to the European Committee on Antimicrobial Susceptibility Testing (EUCAST) clinical breakpoints (2019, v 9.0) [7], to all antimicrobial agents tested except imipenem-relebactam, meropenem-vaborbactam, and colistin. CZA MICs (tested with a fixed avibactam concentration of 4 mg/L) ranged from 32 to 64 mg/L (Supplementary Table 1). PCR and sequencing analysis [8], showed that all isolates harboured *bla*_KPC-2_ as the sole carbapenemase gene. In addition to KPC-2, all eight isolates produced sulfhydryl reagent variable β-lactamase (SHV)-11, OXA-10, Temoniera β-lactamase (TEM)-1 and an extended spectrum β-lactamase (ESBL) of VEB-type. Isolate KP121 produced VEB-14 (T216del, per Ambler numbering scheme) [9]. All other isolates produced a novel VEB-1 variant carrying a substitution of lysine by an arginine at position 237 (K234R, per Ambler numbering scheme), due to nt A710G substitution (Supplementary Table 1). The mutant *bla*_VEB-K237R_ sequence was assigned the novel allele VEB-25 (GenBank accession number: MN853159). Further acquired resistance genes justifying the resistance phenotype of studied strains are presented in Supplementary Table 1.

Whole genome sequencing (WGS) analysis, performed as described previously [10], revealed that both *bla*_VEB-14_ and *bla*_VEB-25_ were carried on conjugative plasmids of IncA/C2 incompatibility group. *bla*_VEB14 _and *bla*_VEB-25_ were present as the first gene cassette of an integron that also included an array of *aadB*, *arr2*, *cmlA1*, *bla*_OXA-10_, and *aadA1* cassettes. An insertion sequence (IS)*1999* was located upstream of both *bla*_VEB_-variants providing a strong promoter for *bla*_VEB_ expression. The two whole genome sequences were deposited in the Sequence Read Archive (SRA) under the following accession numbers: PRJNA602658 (for the *bla*_VEB-14 _harbouring strain) and PRJNA602657 (for the strain with *bla*_VEB-25_).

PFGE analysis classified the studied isolates in two pulsotypes [8], with < 80% Dice similarity index. The first pulsotype included only the KP121, isolated in November 2018, while all the strains isolated in the period September–October 2019, belonged to one clone (second pulsotype). Based on WGS [10], KP121 belonged to sequence type (ST)39 and capsular type KL23 (*wzc*: 24; *wzi*: 83), while the outbreak strain belonged to clonal lineage ST147 and capsular type KL64 (*wzc*: 64; *wzi*: 64).

The ST147 outbreak strain, which was similar to one of the strains reported recently from another hospital in Athens [5], harboured a non-functional porin OmpK35 due to a premature stop codon at position 173, while ST39 isolate (KP121) had an intact OmpK35. OmpK36_v3 variant, previously associated with ST147 *K. pneumoniae* isolates from Greece [11], was present in both ST147 and ST39 isolates. OmpK36_v3 harbours a duplication of two amino acids, Gly134–Asp135, located at the conserved loop L3, which contributes to high-level resistance to carbapenems [11]. However, OmpK35 and OmpK36 are not the primary pathways for avibactam into the cell of *K. pneumoniae* [12].

*bla*_VEB_-harbouring plasmids from the ST39 (KP121), and the ST147 outbreak strain (KP67585) were transferred by conjugation to rifampicin-resistant *Escherichia coli* RC85 R K12. The susceptibility profile of the transconjugants (RC85/pl121 and RC85/pl67585) is shown in Table 1. The susceptibility profile of a previously studied transconjugant, RC85/pl52, harbouring *bla*_VEB-1_ is included in Table 1 for comparison. Transconjugants were resistant to β-lactams, aminoglycosides, trimethoprim/sulfamethoxazole and chloramphenicol (Table 1). PCR and sequencing confirmed the presence of *bla*_VEB_, *bla*_OXA-10,_ *bla*_TEM-1B_ and *rmtB1* and the absence of *bla*_KPC-2_ in all transconjugants. CZA MICs determined by Liofilchem MIC Test Strips, differed between the transconjugants depending on the VEB-variant they produced. RC85/pl52 harbouring *bla*_VEB-1_ exhibited ceftazidime MIC of 512 mg/L, which was reduced to 0.25 mg/L (2,048-fold reduction) in the presence of avibactam. Transconjugant RC85/pl67585 carrying *bla*_VEB-25_, exhibited similar MIC to ceftazidime (512 mg/L), which was reduced to 16 mg/L by avibactam (32-fold reduction). Transconjugant RC85/pl121 carrying *bla*_VEB-14_, exhibited higher MIC to ceftazidime (4,096 mg/L) and to CZA (256 mg/L) (16-fold reduction by avibactam).

## Epidemiological investigation

The eight patients found with a CZA-resistant KPC producing *K. pneumoniae* comprised five men and three women. Possible chains of transmission were investigated but no common source was identified between patients in ICU-1 and ICU-2. All colonised or infected patients had a long period of ICU stay (median: 48 days; range: 11–56 days) before colonisation (Figure). All patients were originally hospitalised in the index hospital with the exception of one patient (KP368), colonised with the outbreak strain, who had been hospitalised in another hospital for 24 hours. None had been previously received CZA, however all patients had been pre-treated or were on β-lactam therapy during hospitalisation in the ICU (Table 2). Only the patient with KP121 had received CZA before colonisation but he represented an independent case not related to the outbreak. Three patients developed an infection: two catheter-related bloodstream infections (catheter culture revealed the same pathogen in both cases) and one ventilator-associated pneumonia. The salvage therapeutic regimens were chosen based on in vitro data showing synergy of the combinations used, all including CZA, the rationale being that avibactam inactivates the class A, C, or D β-lactamases and restores susceptibility to aztreonam or meropenem [13-15]. The triple combination was successful in two of the cases at Day 14, while the combination of CZA and meropenem was reported as failure in the remaining case (Table 2); in terms of all-cause mortality, by Day 28 all infected patients had died.

**Figure:**
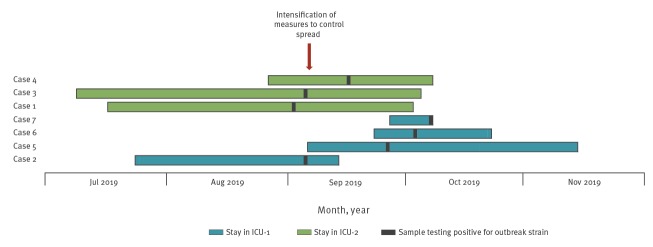
Timeline of the hospital outbreak of**sequence type (ST)147 *Klebsiella pneumoniae* harbouring a Vietnamese extended-spectrum β-lactamase-25, Greece, 2019 (n = 7 outbreak cases)

Cases 1, 2, 3, 4, 5, 6 and 7 correspond to patients 2, 3, 4, 5, 6, 7 and 8 respectively in Table 2.

Intensified measures included the requirement of wearing gloves and a gown upon entering the case’s room, minimal transport of this patient and, if so, prior advising the receiving unit so that necessary precautions could be taken in advance, dedicated use of noncritical items and having dedicated infection control nurses inspecting the implementation of infection control measures, isolation of colonised patients in single rooms and strict hand hygiene practices.

## Discussion

CZA demonstrates high in vitro activity against non-metallo-β-lactamase-producing *K. pneumoniae* strains in Greece [8]. To date, there are two reports on sporadic cases of CZA resistant *K. pneumoniae* from Greece, due to the production of KPC-23 [10], or VEB-25 [5]. In the present study, we provide further evidence that CZA resistance may emerge through evolution of *bla*_VEB-1_ with the detection of a *bla*_VEB-25_-harbouring strain causing a hospital outbreak and another strain endowed with *bla*_VEB-14_ in a single hospitalised patient. Both VEB-14 and VEB-25 variants exhibit decreased inactivation by avibactam.

The *K. pneumoniae* isolates presented in this study produced KPC-2, VEB-14 or VEB-25, OXA-10, TEM-1B and the chromosomally encoded SHV-11 β-lactamases. The transconjugants carried *bla*_OXA-10_, *bla*_TEM-1B_, and *bla*_VEB-1_, or *bla*_VEB-14_ or *bla*_VEB-25_. Avibactam appeared to reduce ceftazidime MICs to a lesser extent in the presence of VEB-14 (16-fold reduction) or VEB-25 (32-fold reduction) than in the presence of VEB-1 (2,048-fold reduction).

VEB-25, which was recently described [5], harbours a substitution of lysine by an arginine at position 237 (K234R, per Ambler numbering scheme), due to nt A710G substitution, while VEB-14 [9], has a threonine deletion at position 217 (T216del, per Ambler numbering scheme).

K234 residue is highly conserved among class A β-lactamases (Supplementary Figure 1), forming strong hydrogen bonds with the sulphate group of avibactam [9]. We hypothesise that K234R substitution resulted in a structural change that attenuated avibactam's inhibitory effect by disrupting its ability to bind at the active site, thereby causing resistance. According to Papp-Wallace et al., residue K234 contributes notably to the inactivation of KPC-2 by avibactam [16], while substitution K234R in SHV enzymes has been reported to lead to resistance to inhibitors [17-20].

T216 residue, although in close proximity, has no direct interaction with avibactam [9]. In an *E. coli* DH5a strain carrying isogenically expressed VEB-14, avibactam reduced ceftazidime MIC from 64 to 1 mg/L, and VEB-14 was inhibited by avibactam in a concentration-dependent manner [9]. We hypothesise that in KP121, CZA resistance was due to increased expression of VEB-14 due to an IS*1999* located upstream of the gene.

Previous epidemiological analyses (data not shown) indicated that a conjugative plasmid co-harbouring *bla*_VEB-1_, *bla*_OXA-10_, *bla*_TEM-1B_ and *rmtB* is present in 8% of all *bla*_KPC_-positive isolates in Greece [21]. This already established vehicle can potentially enhance dissemination of VEB-mediated CZA resistance in Greek hospitals. The emergence of CZA resistance dramatically limits treatment options against carbapenemase-producing Enterobacterales. In this report, infected patients received salvage combination treatment, which was successful in two of the three cases.

Limitations of this study include the lack of environmental sampling during the outbreak investigation, which could have revealed potential environmental sources, and the lack of biochemical studies of VEB-14 and VEB-25 enzymes, which could have further elucidated the basis of the resistance phenotype.

In conclusion, we have shown that alterations in the ESBL VEB-1 enzyme can significantly reduce CZA susceptibility in *K. pneumoniae* co-producing KPC-2 and emergence of this resistance mechanism was independent from previous CZA exposure. Further biochemical studies are needed to reveal the basis of the resistance phenotype, conferred by the two VEB-variants. The rigorous implementation of hospital infection control precautions resulted in successful containment of the outbreak, highlighting the importance of early awareness in the fight against antimicrobial resistance.

## Supplementary Data

875000 Supplementary Material
