# Supplementary material for: Outbreak of KPC-2-producing Klebsiella pneumoniae endowed with ceftazidime-avibactam resistance mediated through a VEB-1-mutant (VEB-25), Greece, September to October 2019
Source: Euro Surveill. 2020 Jan 23;25(3):2000028. doi: 10.2807/1560-7917.ES.2020.25.3.2000028 (PMC6988274; doi:10.2807/1560-7917.ES.2020.25.3.2000028)
Supplement: Supplementary Material [file 2000028_GALANI_Supplementary_Material.pdf]

Supplementary Table S1. Antimicrobial susceptibility and genotypic characteristics of CZA-resistant *Klebsiella pneumoniae* strains isolated from the eight patients

"This supplementary material is hosted by Eurosurveillance as supporting information alongside the article [Outbreak of KPC-2-producing *Klebsiella pneumoniae* endowed with ceftazidime-avibactam resistance mediated through a VEB-1-mutant (VEB-25), Greece, September to November 2019], on behalf of the authors, who remain responsible for the accuracy and appropriateness of the content. The same standards for ethics, copyright, attributions and permissions as for the article apply. Supplements are not edited by Eurosurveillance and the journal is not responsible for the maintenance of any links or email addresses provided therein."

**Case**	**1**	**2**		**3**		**4**	**5**	**6**		**7**	**8**
**Isolate**	**KP121**	**KP370**	**KP67585**	**KP374**	**KP76706**	**KP368**	**KP501**	**KP687**	**KP4969**	**KP785**	**KP842**
**Source**	rectal	rectal	blood	rectal	VAP	rectal	rectal	rectal	blood	rectal	rectal
**PFGE**	Pulsotype I	Pulsotype II		Pulsotype II		Pulsotype II	Pulsotype II	Pulsotype II		Pulsotype II	Pulsotype II
**MLST-type**	ST39	ND	ST147	ST147	ND	ST147	ST147	ST147	ND	ST147	ST147
**KPC-type**	KPC-2	KPC-2	KPC-2	KPC-2	KPC-2	KPC-2	KPC-2	KPC-2	KPC-2	KPC-2	KPC-2
**Ampicillin/Sulbactam**	>16	>16	>16	>16	>16	>16	>16	>16	>16	>16	>16
**Piperacillin/Tazobactam**	>64	>64	>64	>64	>64	>64	>64	>64	>64	>64	>64
**Cefoxitin**	>32	>32	>32	>32	>32	>32	>32	>32	>32	>32	>32
**Ceftazidime**	2048	1024	1024	1024	1024	512	1024	512	1024	1024	1024
**Ceftazidime/Avibactam**	64	64	64	32	32	64	64	64	64	32	64
**Ceftriaxone**	>32	>32	>32	>32	>32	>32	>32	>32	>32	>32	>32
**Cefepime**	>32	>32	>32	>32	>32	>32	>32	>32	>32	>32	>32
**Aztreonam**	>32	>32	>32	>32	>32	>32	>32	>32	>32	>32	>32
**Imipenem**	64	32	32	32	32	32	32	8	16	32	32
**Imipenem/Relebactam**	0.5	0.5	0.5	0.5	0.5	0.5	0.5	0.25	0.25	0.12	0.25
**Meropenem**	>64	64	64	64	64	64	32	16	16	16	16
**Meropenem/Vaborbactam**	0.5	0.25	0.25	0.125	0.125	0.125	0.25	0.125	0.125	0.125	0.125
**Amikacin**	>32	>32	>32	>32	>32	>32	>32	>32	>32	>32	>32
**Gentamicin**	>8	>8	>8	>8	>8	>8	>8	>8	>8	>8	>8
**Ciprofloxacin**	>2	>2	>2	>2	>2	>2	>2	>2	>2	>2	>2
**Levofloxacin**	>4	>4	>4	>4	>4	>4	>4	>4	>4	>4	>4
**Tigecycline**	>4	>4	>4	4	4	>4	>4	2	2	2	2
**Fosfomycin**	64	128	128	64	64	128	128	>128	>128	64	>128
**Colistin**	2	64	64	128	128	128	64	2	16	8	1
**Trimethoprim/Sulfamethoxazole** [a]	>8	>8	>8	>8	>8	>8	>8	>8	>8	>8	>8
**Other β-lactamase genes**	$bla_{\text{SHV-11}}$, **$bla_{\text{VEB-14}}$**, $bla_{\text{OXA-10}}$, $bla_{\text{TEM-1B}}$	bla_{SHV}-like, **$bla_{\text{VEB-25}}$**, $bla_{\text{OXA-10}}$-like, bla_{TEM}-like,	$bla_{\text{SHV-11}}$, **$bla_{\text{VEB-25}}$**, $bla_{\text{OXA-10}}$, $bla_{\text{TEM-1B}}$	$bla_{\text{SHV-11}}$, **$bla_{\text{VEB-25}}$**, $bla_{\text{OXA-10}}$, $bla_{\text{TEM-1B}}$	bla_{SHV}-like, **$bla_{\text{VEB-25}}$**, $bla_{\text{OXA-10}}$-like, bla_{TEM}-like,	bla_{SHV}-like, **$bla_{\text{VEB-25}}$**, $bla_{\text{OXA-10}}$-like, bla_{TEM}-like,	bla_{SHV}-like, **$bla_{\text{VEB-25}}$**, $bla_{\text{OXA-10}}$-like, bla_{TEM}-like,	bla_{SHV}-like, **$bla_{\text{VEB-25}}$**, $bla_{\text{OXA-10}}$-like, bla_{TEM}-like,	bla_{SHV}-like, **$bla_{\text{VEB-25}}$**, $bla_{\text{OXA-10}}$-like, bla_{TEM}-like,	$bla_{\text{SHV-11}}$, **$bla_{\text{VEB-25}}$**, $bla_{\text{OXA-10}}$, $bla_{\text{TEM-1B}}$	bla_{SHV}-like, **$bla_{\text{VEB-25}}$**, $bla_{\text{OXA-10}}$-like, bla_{TEM}-like,
**Other resistance determinants**	*aac(3)-IId, aadA1, aadA2, ant(2'')-Ia, ant(3'')-Ia,*	ND	*aadA1, aadA2, ant(2'')-Ia, aph(3'')-Ib, aph(3')-Ia,*	*aadA1, aadA2, ant(2'')-Ia, aph(3'')-Ib, aph(3')-Ia,*	ND	ND	ND	ND	ND	*aadA1, aadA2, ant(2'')-Ia, aph(3'')-Ib, aph(3')-Ia,*	ND

		aph(3'')-Ib, aph(3')-Ia, aph(6)-Id, rmtB, fosA, arr-2, cmlA1, dfrA12, dfrA14, dfrA23, tet(A), tet(G), tet(M), sul1, sul2, sul3		*aph(6)-Id, rmtB, fosA, arr-2, cmlA1, dfrA12, tet(A), tet(G), sul1, sul2*	*aph(6)-Id, rmtB, fosA, arr-2, cmlA1, dfrA12, tet(A), tet(G), sul1, sul2*						*aph(6)-Id, rmtB, fosA, arr-2, cmlA1, dfrA12, tet(A), tet(G), sul1, sul2*	
**Major porin mutation**	**OmpK35**	WT	ND	PSC_aa173	PSC_aa173	ND	ND	ND	ND	ND	PSC_aa173	ND
	**OmpK36**	v3 variant	ND	v3 variant	v3 variant	ND	ND	ND	ND	ND	v3 variant	ND
	**OmpK37**	PSC_aa251	ND	WT	WT	ND	ND	ND	ND	ND	WT	ND

[a] Trimethoprim-sulfamethoxazole in the ratio 1:19. MICs are expressed as the trimethoprim concentration.

WT, wild-type; ND, not determined; PSC, premature stop codon; OmpK35-WT, GU460162; OmpK36_v3, JQ781655; OmpK37-WT, WP_002902433; PSC_aa173, premature stop codon at amino acid 173; PSC_aa251, premature stop codon at amino acid 251

Supplementary Figure S1. Alignment of the amino acid sequences of VEB-1, VEB-14 and VEB-25 with those of class A-lactamases PER-1, TEM-1, SHV-1, CTX-M-1, GES-1 and KPC-2.

"This supplementary material is hosted by Eurosurveillance as supporting information alongside the article [Outbreak of KPC-2-producing Klebsiella pneumoniae endowed with ceftazidime-avibactam resistance mediated through a VEB-1-mutant (VEB-25), Greece, September to November 2019], on behalf of the authors, who remain responsible for the accuracy and appropriateness of the content. The same standards for ethics, copyright, attributions and permissions as for the article apply. Supplements are not edited by Eurosurveillance and the journal is not responsible for the maintenance of any links or email addresses provided therein."

```
               1   10                 20          30        40        50
               ↓   ↓                  ↓           ↓         ↓         ↓
PER-1          --MNVIIKAVVTASTLLMVSFSSFETSAQ---SPLLKEQIESIVIGKKATVGVAVWGPDD
VEB-14         --MKIVKRILL---VLLSLFFTIVYSNAQ---TDNLTLKIENVLKAKNARIGVAIFNSNE
VEB-1          --MKIVKRILL---VLLSLFFTIVYSNAQ---TDNLTLKIENVLKAKNARIGVAIFNSNE
VEB-25         --MKIVKRILL---VLLSLFFTIVYSNAQ---TDNLTLKIENVLKAKNARIGVAIFNSNE
GES-1          --MRFIHA----------LLLAGIAHSAYASEKLTFKTDLEKLEREKAAQIGVAIVDPQ-
TEM-1          MSIQHFRV-------ALIPFFAAFCLPVF--AHPETLVKVKDAEDQLGARVGYIELDLNS
SHV-1          --MRYIRL-------CIISLLATLPLAVH--ASPQPLEQIKLSESQLSGRVGMIEMDLAS
CTX-M-1        MVKKSLRQFTLMATATVTLLLGSVPLYAQ---TADVQQKLAELERQSGGRLGVALINTAD
KPC-2          --MSLYRRLVLLS--CLSWPLAGFSATAL---TNLVAEPFAKLEQDFGGSIGVYAMDTGS

                                                                  Box-I

                     60        70        80        90        100        110
                     ↓         ↓         ↓         ↓         ↓          ↓
PER-1          LEP-LLINPFEKFPMQSVFKLHLAMLVLHQVDQGKLDLNQTVIVNRAKVLQNTWAPIMKA
VEB-14         KDT-LKINNDFHFPMQSVMKFPIALAVLSEIDKGNLSFEQKIEITPQDLLPKTWSPIKEE
VEB-1          KDT-LKINNDFHFPMQSVMKFPIALAVLSEIDKGNLSFEQKIEITPQDLLPKTWSPIKEE
VEB-25         KDT-LKINNDFHFPMQSVMKFPIALAVLSEIDKGNLSFEQKIEITPQDLLPKTWSPIKEE
GES-1          GEIVAGHRMAQRFAMCSTFKFPLAALVFERIDSGTERGDRKLSYGPDMIV--EWSPATER
TEM-1          GKILESFRPEERFPMMSTFKVLLCGAVLSRVDAGQEQLGRRIHYSQNDLV--EYSPVTEK
SHV-1          GRTLTAWRADERFPMMSTFKVVLCGAVLARVDAGDEQLERKIHYRQQDLV--DYSPVSEK
CTX-M-1        NSQ-ILYRADERFAMCSTSKVMAVAAVLKKSESEPNLLNQRVEIKKSDLV--NYNPIAEK
KPC-2          GAT-VSYRAEERFPLCSSFKGFLAAAVLARSQQQAGLLDTPIRYGKNALV--PWSPISEK
                               *  *                                  **
                              Box-II                               Box-III Box-IV

                       120       130       140       150       160
                       ↓         ↓         ↓         ↓         ↓
PER-1          YQGDEFSVPVQQLLQYSVSHSDNVACDLLFELVGGPAALHDYIQSMGIKETAVVANEAQM
VEB-14         FPNG-TTLTIEQILNYTVSESDNIGCDILLKLIGGTDSVQKFLNANHFTDISIKANEEQM
VEB-1          FPNG-TTLTIEQILNYTVSESDNIGCDILLKLIGGTDSVQKFLNANHFTDISIKANEEQM
VEB-25         FPNG-TTLTIEQILNYTVSESDNIGCDILLKLIGGTDSVQKFLNANHFTDISIKANEEQM
GES-1          FLAS-GHMTVLEAAQAAVQLSDNGATNLLLREIGGPAAMTQYFRKIGDSVSRLDRKEPEM
TEM-1          HLTD--GMTVRELCSAAITMSDNTAANLLLTTIGGPKELTAFLHNMGDHVTRLDRWEPEL
SHV-1          HLAD--GMTVGELCAAAITMSDNSAANLLLATVGGPAGLTAFLRQIGDNVTRLDRWETEL
CTX-M-1        HVDG--TMSLAELSAAALQYSDNVAMNKLISHVGGPASVTAFARQLGDETFRLDRTEPTL
KPC-2          YLTT--GMTVAELSAAAVQYSDNAAANLLLKELGGPAGLTAFMRSIGDTTFRLDRWELEL
                                   * *                                     *
                                                                         Box-V

               170       180       190         200       210       220
               ↓         ↓         ↓           ↓         ↓         ↓
PER-1          HADDQVQYQNWTSMKGAAEILK-KFEQK-TQLSETSQALLWKWMVETTTGPERLKGLLPA
VEB-14         HKDWNTQYQNWATPTAMNKLLIDTYNNKNQLLSKKSYDFIWKIMRET-TGSNRLKGQLPK
VEB-1          HKDWNTQYQNWATPTAMNKLLIDTYNNKNQLLSKKSYDFIWKIMRETTTGSNRLKGQLPK
VEB-25         HKDWNTQYQNWATPTAMNKLLIDTYNNKNQLLSKKSYDFIWKIMRETTTGSNRLKGQLPK
GES-1          GDNTPGDLRDTTTPIAMARTVAKVLY--GGALTSTSTHTIERWLIGNQTGDATLRAGFPK
TEM-1          NEAIPNDERDTTMPAAMATTLRKLLT--GELLTLASRQQLIDWMEADKVAGPLLRSALPA
SHV-1          NEALPGDARDTTTPASMAATLRKLLT--SQRLSARSQRQLLQWMVDDRVAGPLIRSVLPA
CTX-M-1        NTAIPGDPRDTTSPRAMAQTLRNLTL--GKALGDSQRAQLVTWMKGNTTGAASIQAGLPA
KPC-2          NSAIPGDARDTSSPRAVTESLQKLTL--GSALAAPQRQQFVDWLKGNTTGNHRIRAAVPA
               *                                                  *   #
                                                       Box-VI
```

```
                230       240           250      260       270       280
                 ↓         ↓             ↓        ↓         ↓         ↓
PER-1         GTVVAHKTGTSGIKAGKTAATNDLGIILLPDGRPLLVAVFVKDSAESSRTNEAIIAQVAQ
VEB-14        NTIVAHKTGTSGINNGIAAATNDVGVITLPNGQLIFISVFVAESKETSEINEKIISDIAK
VEB-1         NTIVAHKTGTSGINNGIAAATNDVGVITLPNGQLIFISVFVAESKETSEINEKIISDIAK
VEB-25        NTIVAHRTGTSGINNGIAAATNDVGVITLPNGQLIFISVFVAESKETSEINEKIISDIAK
GES-1         DWVVGEKTGTCANG-----GRNDIGFFKAQE-RDYAVAVYTTAPKLSAVERDELVASVGQ
TEM-1         GWFIADKSGA-GER----GSRGIIAALGPDGKPSRIVVIYTTGSQATMDERNRQIAEIGA
SHV-1         GWFIADKTGA-GER----GARGIVALLGPNNKAERIVVIYLRDTPASMAERNQQIAGIGA
CTX-M-1       SWVVGDKTGS-GDY----GTTNDIAVIWPKDRAPLILVTYFTQPQPKAESRRDVLASAAK
KPC-2         DWAVGDKTGTCGVY----GTANDYAVVWPTGRAPIVLAVYTRAPNKDDKHSEAVIAAAAR
                    ** *
                  Box-VII

                   290
                    ↓
PER-1         TAYQFELKKLSALSPN
VEB-14        ITWNYYLNK-------
VEB-1         ITWNYYLNK-------
VEB-25        ITWNYYLNK-------
GES-1         VITQLILSTDK-----
TEM-1         SLIKHW----------
SHV-1         ALIEHWQR--------
CTX-M-1       IVTNGL----------
KPC-2         LALEGLGVNGQ-----
```

Dashes indicate gaps within the alignment. Standard numbering for class A enzymes according to Ambler [1] is indicated. The Roman numerals below the shaded boxes indicate conserved regions within the class A-lactamases according to Joris et al. [2]. Asterisks indicate the 12 residues line the avibactam binding pocket as reported by Lahiri et al for CTX-M-1[3]. Asterisks in bold indicate while in non-bold residues that differ from CTX-M-15 although conserved among VEB enzymes [4]. Hash mark indicates an additional residue (R220) that contributes significantly to the mechanism of avibactam inactivation of KPC-2 [5]. This figure was produced by Clustal Omega multiple sequence alignment program (1.2.4).

References

1. Ambler RP. The structure of β-lactamases. Philos Trans R Soc Lond B Biol Sci 1980; 289(1036): 321–331. https://doi.org/10.1098/rstb.1980.0049
2. Joris B, Ledent P, Dideberg O, Fonze´ E, Lamotte-Brasseur J, Kelly JA, et al. Comparison of the sequences of class A β-lactamases and of the secondary structure elements of penicillin-recognizing proteins. Antimicrob Agents Chemother 1991; 35(11): 2294–2301. https://doi.org/10.1128/aac.35.11.2294
3. Lahiri SD, Mangani S, Durand-Reville T, Benvenuti M, De Luca F, Sanyal G, et al. Structural insight into potent broad-spectrum inhibition with reversible recyclization mechanism: avibactam in complex with CTX-M-15 and *Pseudomonas aeruginosa* AmpC β-lactamases. Antimicrob Agents Chemother 2013; 57(6): 2496-505. https://doi.org/10.1128/AAC.02247-12
4. Lahiri SD, Alm RA. Identification of Novel VEB β-Lactamase Enzymes and Their Impact on Avibactam Inhibition. Antimicrob Agents Chemother 2016; 60(5): 3183-6. https://doi.org/10.1128/AAC.00047-16
5. Papp-Wallace KM, Winkler ML, Taracila MA, Bonomo RA. Variants of β-lactamase KPC-2 that are resistant to inhibition by avibactam. Antimicrob Agents Chemother 2015; 59(7): 3710-7. https://doi.org/10.1128/AAC.04406-14
